# The Relationship Between MOTS-c K14Q Polymorphism and Sarcopenia, Blood Lipids, and Mental Health in Older Korean Adults

**DOI:** 10.3390/biomedicines12102384

**Published:** 2024-10-18

**Authors:** Shinuk Kim

**Affiliations:** Gyedang College of General Education, Sangmyung University, Cheonan 31066, Chungcheongnam-do, Republic of Korea; kshinuk@gmail.com; Tel.: +82-41-550-5452

**Keywords:** body composition, handgrip strength, mitochondrial gene, older adult, sarcopenia

## Abstract

**Background/objectives:** An East Asian-specific 1382A>C polymorphism in the mitochondrial open reading frame of the 12S rRNA type-c results in an amino acid substitution from Lys (K) to Gln (Q) at the 14th amino acid residue. This study investigated the association between m.1382A>C polymorphism and sarcopenia, blood lipids, and mental health in older Korean adults. **Methods:** The study included 683 community-dwelling Korean adults (345 men and 338 women) aged 65 years and older. The m.1382A>C polymorphism was genotyped with a 7500 real-time PCR system. Handgrip strength (HGS) was measured, and appendicular skeletal muscle (ASM) mass was calculated. Demographics, blood lipids, falling risk, nutritional intake, cognition function, and depression were additionally measured. **Results:** Men carrying the C allele had significantly higher ASM (21.6 ± 3.0 vs. 19.5 ± 2.2 kg, *p* = 0.018), ASM/height^2^ (7.76 ± 0.76 vs. 7.14 ± 0.62 kg/m^2^, *p* = 0.012), lean mass (53.3 ± 6.2 vs. 46.5 ± 4.0 kg, *p* < 0.001), left HGS (33.3 ± 5.0 vs. 28.9 ± 4.0 kg, *p* = 0.010), and right HGS (35.6 ± 5.3 vs. 30.9 ± 4.3 kg, *p* = 0.009) than men carrying the A allele. The genotype differences in ASM (*p* = 0.017), ASM/height^2^ (*p* = 0.011), lean mass (*p* < 0.001), left HGS (*p* = 0.010), and right HGS (*p* = 0.009) remained significant even after adjusting for all measured covariates. By contrast, no significant differences in other measured parameters were found between women carrying the A and C alleles. **Conclusions:** Our study findings indicate that the m.1382A>C polymorphism may be used as a genetic biomarker of age-related sarcopenia in older Korean men.

## 1. Introduction

The 12S ribosomal RNA (rRNA) region encodes the mitochondrial open reading frame of the 12S rRNA type-c (MOTS-c), a 16-amino acid mitochondria-derived peptide [1]. MOTS-c is transported to the nucleus in response to metabolic stress or exercise, where it promotes cellular homeostasis by regulating nuclear gene expression [2]. MOTS-c primarily acts via the folate 5-aminoimidazole-4-carboxamide riboside (AICAR)-AMP-activated protein kinase (AMPK) pathway, which regulates skeletal muscle fiber type and insulin sensitivity [3,4], inhibits high-fat-diet-induced weight gain and hepatic steatosis [5], slows aging processes [1], and reduces inflammation [1].

Mitochondrial DNA (mtDNA), the DNA found inside the cellular organelles, is polymorphic, and the mtDNA polymorphism is associated with alterations in mitochondrial functions [1,6]. Recent rodent and human studies by a group of Japanese researchers have focused on the mtDNA polymorphism m.1382A>C in the MOTS-c cording region because it causes an amino acid substitution from Lys (K) to Gln (Q) at the 14th amino acid residue (MOTS-c K14Q), which is specific to the Northeast Asian population [3,7,8]. MOTS-c administration improves insulin sensitivity in skeletal muscle and glucose homeostasis by inducing an exercise-mimicking effect [9], indicating its benefits for various metabolic diseases such as obesity and type 2 diabetes (T2D). A meta-analysis of three cohort studies, including the Japan Multi-Institutional Collaborative Cohort (J-MICC) Study, the Multiethnic Cohort (MEC) study, and the Tohoku Medical Megabank Project (TMM), found that men who were C allele carriers of the m.1382A>C polymorphism had a higher risk of visceral obesity and T2D [5]. An additional analysis of the J-MICC study revealed that C allele carriers with low physical activity had a 65% higher prevalence of T2D than A allele carriers with low physical activity, indicating a kinesio–genomic interaction in disease susceptibility [2]. In addition to metabolic disorders, the association between the m.1382 A>C polymorphism and sarcopenia and physical performance has been reported. In an animal study, for example, MOT-c administration prevented muscle atrophy in diet-induced obese mice by lowering plasma myostatin levels and myostatin expression in skeletal muscle [7]. MOTS-c administration also improved running time and distance in wild-type mice [2,10]. The C allele of the m.1382A>C polymorphism is associated with more fast-twitch fibers than the A allele. Gender-specific analysis revealed that men carrying the C allele had higher peak torques of leg flexion and extension and a higher sprint/power athletic performance than men carrying the A allele [3].

Sarcopenia, defined as a normal, progressive decline in muscle mass, strength, and function, is an unavoidable consequence of aging [11]. Sarcopenia is linked to an increased risk of geriatric health conditions, including falls, fractures, frailty, cachexia, and osteoporosis [12]. However, the prevalence of sarcopenia and its impact on health can differ depending on race [13], hormones [14], lifestyle [15], socioeconomic background [16], genetics [17], and other factors [18]. Furthermore, recent research indicates that the East Asian-specific K14Q polymorphism may contribute to individual differences in sarcopenia [7,8]. No previous study has investigated the genetic susceptibility of the m.1382 A>C polymorphism to age-related sarcopenia and other health conditions in Korean populations. This study aimed to investigate the diagnostic value of the MOTS-c K14Q polymorphism in predicting the risk of sarcopenia, as well as other health issues, including falling, dyslipidemia, cognition, and depression, in a sample of older Korean adults.

## 2. Materials and Methods

### 2.1. Participants

**Figure 1:**
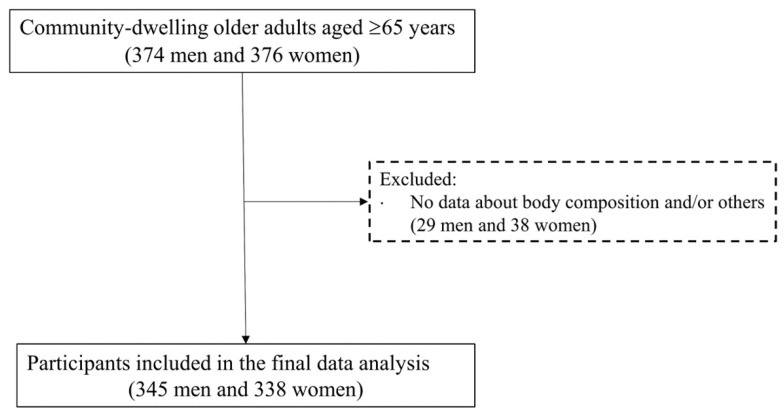
Selection of study participants.

In a cross-sectional study design, as shown in Figure 1, we initially recruited 750 community-dwelling older adults aged 65 and up (374 men and 376 women) from local communities in Suwon, Republic of Korea. Inclusion criteria included being at least 65 years old and living in a community. Exclusion criteria included any health condition that would severely limit participation in physical and mental health assessments. Those with missing data on body composition and/or other variables (29 men and 38 women) were excluded, leaving 683 participants (345 men and 338 women) for the final analysis. The sample size was calculated with the G*Power software (version 3.1.9.7). Based on our preliminary data (effect size = 0.190) on skeletal muscle index, we calculated that a sample size of 349 participants per gender would provide 85% power with a probability of alpha error of 0.05 to detect a statistically significant difference in the primary outcome between the two alleles. All participants were fully informed of the study objectives, methods, and procedures. They provided informed consent before participating in the study. The study was reviewed and approved by the Institutional Review Board of Human Subjects in accordance with the Declaration of Helsinki (approval number 2018-06-005-003).

### 2.2. Body Composition, Handgrip Strength, Falling Concern, and Balance Confidence

A dual x-ray absorptiometry system (QDR 4500A, Hologic, Waltham, MA, USA) was used to measure total body composition, which included height, weight, muscle mass, and body fat. The BMI was calculated by dividing weight (kg) by height (m^2^). Appendicular skeletal mass (ASM), a proxy biomarker of sarcopenia, was calculated by adding dual x-ray absorptiometry (DEXA)-based muscle mass in the arms and legs. The same certified technician positioned the subjects, performed the scans, and carried out the analysis according to the operator’s manual and standard analysis protocol. Based on ten subjects, the coefficients of variation (CVs) for FM and FFM were 2.5 and 3.1%, respectively. Furthermore, our DXA equipment is calibrated daily with a spine phantom (supplied by the manufacturers) to ensure measurement stability. It is also calibrated weekly with a step phantom to correct for errors such as skin thickness. The waist circumference (WC) was measured at the midpoint of the ribcage and the iliac crest, while the hip circumference (HC) was measured at the widest part of the buttock. The waist-to-hip ratio (WHR) was calculated by dividing WC (cm) by HC. Handgrip strength (HGS) was determined using a digital hand dynamometer (T.K.K 5401, Takei Scientific Instruments Co., Ltd., Tokyo, Japan). In a standing position, each participant tested her or his dominant hand’s maximum handgrip strength twice, with a 30 s rest interval between each measurement. The two attempted values for the right and left hands were combined and expressed as a relative term (kg per body mass index).

The Activities-Specific Balance Confidence (ABC) Scale is a reliable tool for identifying elderly people who are at risk of falling due to neurological disorders [19]. The Korean version of the ABC Scale, a 16-item questionnaire with confidence ratings ranging from 0% (not confident) to 100% (very confident), was used to assess the study participants’ overall confidence. Averaging the ratings yields a total score, with higher scores indicating greater balance confidence. The Korean falling efficacy scale (K-FES) was used to determine fall self-efficacy [20]. Ten items are scored using a 10-point ordinal scale, with a maximum possible score of 100 points. The K-FES items include the following: (1) taking a bath or shower, (2) reaching up to a closet, (3) doing light housekeeping (e.g., cleaning up your nightstand or dresser), (4) walking around the nursing home, (5) getting in and out of bed, (6) getting up at night to use the restroom, (7) getting in and out of a chair, (8) getting dressed and undressed, (9) doing personal grooming (e.g., washing your face, combing your hair), and (10) getting on and off the toilet.

The Korean-Mini-Mental Status Examination (K-MMSE) was used to evaluate cognitive function [21]. Depression was assessed using the 10-item short form of the Center for Epidemiological Studies Depression (CESD-10) scale’s modified Korean version [22]. In addition, covariates included age (in years), education (in years), smoking (past/current smokers vs. non-smokers), number of existing chronic diseases, fall experience, and physical activity. The number of non-communicable chronic disease(s), such as arthritis, stroke, angina, diabetes, chronic lung disease, asthma, depression, hypertension, and others, was self-reported based on a physician’s diagnosis, by answering the following question: have you ever been diagnosed with hypertension (high blood pressure)? The International Physical Activity Questionnaire (IPAQ) was used to track moderate to vigorous physical activity, which was measured in minutes per week and classified as insufficiently or sufficiently active (150 min per week) [23].

### 2.3. Blood Chemistry and Genotyping

Fasting blood glucose (FBG), total cholesterol (TC), triglycerides (TGs), low-density lipoprotein cholesterol (LDL-C), and high-density lipoprotein cholesterol (HDL-C) were measured using an enzymatic calorimeter and a Beckman Coulter. The serum vitamin D level (25-hydroxyvitamin D, 25(OH)D, ng/mL) was determined using a gamma counter (1470 Wizard, Perkin-Elmer, Turku, Finland).

The QIAamp DNA Blood Mini Kit (Qiagen, Hilden, Germany) was used to extract total DNA from venous blood, following the manufacturer’s instructions. The DNA concentration was measured with a Thermo Scientific NanoDrop 8000 UV–Vis spectrophotometer (Thermo Fisher Scientific, DE, USA). DNA samples were kept at 4 °C until used. The m.1382A>C polymorphism (rs111033358) was genotyped with a custom TaqMan^®^ SNP Genotyping Assay and a 7500 real-time PCR system [3]. Genotypes were determined with the LightCycler^®^ 480 SW (Roche Molecular Systems).

### 2.4. Statistics

A quantile–quantile plot was used to confirm the normality of the data distribution before statistical analysis. Descriptive statistics are reported as means ± standard deviations. The m.1382A>C polymorphism’s allele frequencies were compared using the chi-square test. The unpaired *t*-test or Mann–Whitney *U* test was used to determine significant differences in measured variables between genotypes. All statistical analyses were performed with the SPSS statistical software, version 21.0 (SPSS Inc., Chicago, IL, USA). The statistical significance was determined at *p* = 0.05.

## 3. Results

Table 1 shows age, education, smoking, body composition, and number of physician-diagnosed diseases according to the MOTS-c K14Q genotype. Genotype analysis revealed that the C allele frequency was 5.1%, with a similar frequency between men and women (5.5% vs. 4.7%). There were no significant differences in mean age, education, smoking, BMI, WC, WHR, and number of physician-diagnosed diseases according to the m.1382A>C polymorphism. 

**Figure 2:**
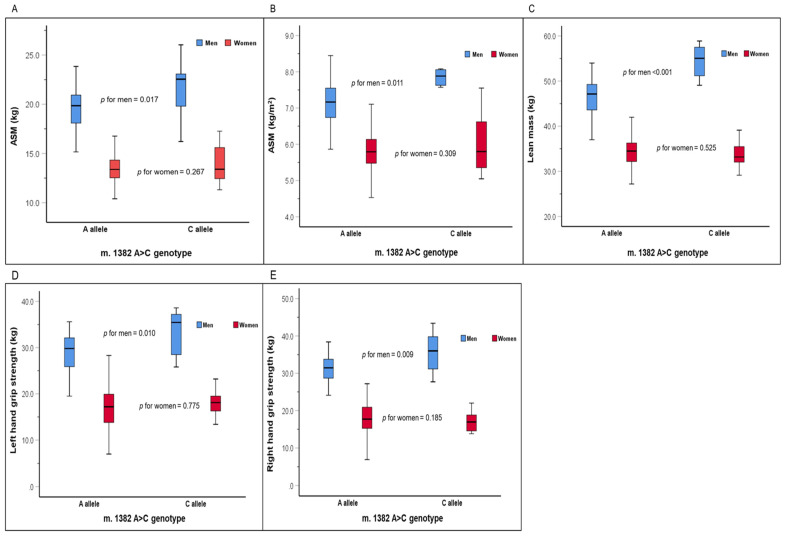
Appendicular skeletal muscle (**A**,**B**), lean mass (**C**), and handgrip strength (**D**,**E**) by the m.1382 A<C genotype. Table 2 represents the comparative statistics of the blood chemistry profiles, health behaviors such as dietary intake and physical activity, fall risk parameters, and mental health parameters according to the MOTS-c K14Q genotype. In the total group, the C allele carrier of the m.1382A>C polymorphism had a significantly higher TC concentration (*p* = 0.014) and LDLC (*p* = 0.038) than the A allele carrier. Furthermore, women with the C allele had lower TC (*p* = 0.005) and LDLC (*p* = 0.030) than those with the A allele. On the other hand, no significant genotype differences were observed in other blood chemistry profiles such as FBG, TG, and HDLC; dietary intake, such as carbohydrates, fats, and proteins; physical activity; fall experience; fall efficacy scale; ABC scale; K-MMSE; and CESD−10, either in the total group or in men or women only.

As shown in Figure 2, there were significant differences in the parameters of sarcopenia (Figure 2A,B), body composition (Figure 2C), and handgrip strength (Figure 2D,E) among people with the m.1382A>C polymorphism. In men, the C allele carriers of the m.1382 A>C polymorphism had significantly higher values in ASM (21.6 ± 3.0 vs. 19.5 ± 2.2 kg, *p* = 0.018), ASM/height^2^ (7.76 ± 0.76 vs. 7.14 ± 0.62 kg/m^2^, *p* = 0.012), lean mass (53.3 ± 6.2 vs. 46.5 ± 4.0 kg, *p* < 0.001), left handgrip strength (33.3 ± 5.0 vs. 28.9 ± 4.0 kg, *p* = 0.010), and right handgrip strength (35.6 ± 5.3 vs. 30.9±4.3 kg, *p* = 0.009) than the A allele carriers. The genotype differences in ASM (Figure 2A, *p* = 0.017), ASM/height^2^ (Figure 2B, *p* = 0.011), lean mass (Figure 2C, *p* < 0.001), left handgrip strength (Figure 2D, *p* = 0.010), and right handgrip strength (Figure 2E, *p* = 0.009) remained significant even after adjusting for all the measured covariates. In women, there were no genotype differences in ASM, ASM/height^2^, lean mass, left handgrip strength, and right handgrip strength.

## 4. Discussion

Sarcopenia has a complex etiology that is attributed to several factors, including oxidative stress, inflammation, apoptosis, and mitochondrial dysfunction, as well as an inadequate diet, sedentary lifestyles, and the interaction of these factors. Studies have investigated the genetic influence on skeletal muscle traits and estimated the heritability of muscle strength ranging from 30% to 85% [24,25]. In this regard, we examined the association between the East Asian-specific K14Q polymorphism and sarcopenia, body composition, and handgrip strength in community-dwelling Korean adults aged 65 years and older. Our study findings showed that older Korean men with the C allele have greater ASM and handgrip strength than older Korean men with the A allele, with no such genotype difference in older Korean women, implying that the m.1382A>C polymorphism may be clinically relevant as a genetic biomarker for age-related sarcopenia.

To the best of our knowledge, we are the first to report that the m.K14Q polymorphism is significantly associated with sarcopenia in older Korean men. Although there is no direct evidence to support the current finding, both animal and human studies have shown that MOTS-c administration prevented fatty acid-induced atrophy in differentiated C2C12 myotubes and inhibited high-fat-diet-induced muscle atrophy in C57BL/6J mice by suppressing systemic and tissue-specific levels of myostatin expression [7]. The K14Q mutation was related to higher expression of myosin-heavy chain isotype expression in human skeletal muscle, as well as higher muscle strength and higher sprint/power athletic performance [8]. Taken together, the findings from the previous studies suggest that the C allele may have a genetic advantage over the A allele in terms of skeletal-muscle-related phenotypes. A genome-wide association study on Korean cohorts showed that ribosomal protein S10 (RPS10), nudix hydrolase 3 (NUDT3), and glycerol-3-phosphate dehydrogenase 1 like (GPD1L) are genetic biomarkers of age-related sarcopenia [26]. Our findings support the application of m.1382A>C polymorphism as a potential genetic biomarker for age-related sarcopenia in older Korean men.

There are some explanations for the link between the C allele of the m.1832A>C polymorphism and muscle biology. Mice treated with the MOTS-c neutralizing antibody had significantly higher protein expression of MHC-IIX via PGC-1α and FOXO1 signaling pathways than control mice [3], as well as a downregulation of skeletal muscle myostatin, a negative regulator of the skeletal muscle. Overexpression and knockout of PGC-1α in mice led to an increase in slow- and fast-twitch fibers, respectively [27]. Furthermore, muscle-specific FOXO1 overexpression reduced gene expression levels associated with slow-twitch fibers [28]. Together, the current and previous studies suggest that the m.1382A>C polymorphism may account for some of the individual variations in sarcopenia parameters observed in East Asian populations such as Japanese and Korean populations. By contrast, the findings from the J-MICC study showed that the C allele carriers interacted with physical inactivity, increasing the risk of T2D [2]. As a result, we cannot rule out the possibility that the kinesio–genomic interaction may also explain the association between the 1382A>C polymorphism and sarcopenia, body composition, and handgrip strength observed in the current study. Additionally, it was interesting to find that only among older women, the C allele carriers had higher TC and LDL-C levels than the A allele carriers. We have no explanation for this gender-specific genotype difference in blood lipid profiles. However, previous studies have reported that the MOTS-c K14Q polymorphism is associated with impaired glucose metabolism [4], diet- and age-induced insulin resistance [29], type 2 diabetes [5], visceral adiposity [5], and inflammation [6]. The atherogenic lipid profiles observed in the C allele carriers suggest that the K14Q polymorphism may serve as a biomarker of gender-specific susceptibility to metabolic complications [30], which should be confirmed in a future large-scale study. 

This study has some limitations. First, the cross-sectional nature of the study precludes any causal explanation for the relationship between the K14Q polymorphism and sarcopenia parameters, which must be confirmed in a prospective large-scale study. Second, we cannot rule out the possibility that the genetic predisposition of the K14Q variant to sarcopenia is influenced by lifestyle factors such as nutrition and exercise [31]. This may provide some explanations for the gender-specific susceptibility of the K14Q variant to sarcopenia, low lean mass, and low handgrip strength observed in this study [5]. An intervention study will be required to investigate the potential effects of the genotype and environmental factors on sarcopenia and other health issues in different populations. 

## 5. Conclusions

In conclusion, the current study’s findings suggest that the m.1382A>C polymorphism could be used as a genetic biomarker to identify age-related loss of muscle mass and strength in Korean older men. This genetic association study could serve as a starting point for determining the relative impact of environmental genetic variants on aging-related health conditions.

## Figures and Tables

**Table 1:** Descriptive statistics of study participants according to MOTS-c K14Q polymorphism.

Variables	Total (*n* = 683)				Men (*n* = 345)				Women (*n* = 338)			
	A Allele (*n* = 648)	C Allele (*n* = 35)	ES	*p*	A Allele (*n* = 326)	C Allele (*n* = 19)	ES	*p*	A Allele (*n* = 322)	C Allele (*n* = 16)	ES	*p*
Age (years)	74.1 ± 6.6	72.5 ± 7.0	0.248	0.239	73.9 ± 5.5	74.0 ± 5.3	−0.015	0.969	74.1 ± 6.7	71.7 ± 5.6	0.361	0.160
Education (years)	7.2 ± 4.4	7.6 ± 3.9	−0.098	0.663	11.3 ± 3.9	8.7 ± 5.5	0.622	0.125	6.4±	7.1±	−0.162	0.553
BMI (kg/m^2^)	24.5 ± 3.1	24.9 ± 3.3	−0.111	0.602	23.8 ± 2.5	24.7 ± 2.2	−0.392	0.301	24.7 ± 3.2	24.9 ± 3.8	−0.081	0.752
WC (cm)	91.3 ± 13.4	91.6 ± 14.6	0.022	0.922	91.7 ± 11.6	99.2 ± 15.1	−0.621	0.105	91.2 ± 13.7	87.8 ± 13.3	0.249	0.363
WHR	0.91 ± 0.07	0.90 ± 0.08	0.199	0.375	0.92 ± 0.05	0.92 ± 0.04	−0.129	0.748	0.91 ± 0.07	0.89 ± 0.09	−0.187	0.201
Smoking, n (%)	271 (41.9)	14 (39.3)	0.159	0.736	246 (75.5)	15 (77.1)	0.225	0.673	29 (8.9)	2 (9.1)	0.027	0.825
# of chronic diseases	3.2 ± 1.7	3.1 ± 1.0	0.084	0.653	2.8 ± 1.1	3.4 ± 1.1	−0.518	0.181	3.3 ± 1.0	3.0 ± 0.9	0.285	0.279

ES: Cohen’s d; BMI, body mass index; WC; waist circumference; WHR, waist-to-hip ratio.

**Table 2:** Blood chemistry, health behaviors, falling risk, and mental health parameters according to MOTS-c K14Q polymorphism.

Variables	Total (*n* = 683)				Men (*n* = 345)				Women (*n* = 338)			
	A Allele (*n* = 648)	C Allele (*n* = 35)	ES	*p*	A Allele (*n* = 326)	C Allele (*n* = 19)	ES	*p*	A Allele (*n* = 322)	C Allele (*n* = 16)	ES	*p*
**Blood chemistry profiles**												
FBG (mg/dL)	114.8 ± 25.1	117.3 ± 34.9	−0.097	0.644	118.8 ± 31.8	103.9 ± 7.3	0.021	0.193	114.1 ± 23.5	124.1 ± 41.2	−0.406	0.114
TC (mg/dL)	158.2 ± 37.4	177.5 ± 36.9	0.522	0.014	172.9 ± 35.5	172.0 ± 40.0	0.026	0.946	151.3 ± 35.2	178.4 ± 37.2	0.729	0.005
TG (mg/dL)	128.1 ± 59.3	108.5 ± 41.6	0.335	0.112	137.4 ± 73.5	114.6 ± 50.7	0.318	0.400	125.3 ± 56.1	105.4 ± 37.7	0.377	0.142
HDLC (mg/dL)	51.8 ± 13.5	50.2 ± 12.1	0.121	0.566	48.5 ± 12.0	53.6 ± 11.5	−0.429	0.258	52.4 ± 13.7	48.4 ± 12.4	0.292	0.255
LDLC (mg/dL)	86.4 ± 28.7	100.1 ± 33.6	0.411	0.038	97.0 ± 31.3	95.5 ± 28.2	0.049	0.897	81.8 ± 28.7	100.7 ± 34.0	0.558	0.030
Vitamin D (ng/mL)	19.5 ± 9.8	19.4 ± 8.4	0.011	0.958	19.4 ± 8.7	20.6 ± 6.9	−0.144	0.703	19.5 ± 10.1	18.8 ± 9.2	0.073	0.775
**Nutritional intake**												
PRO (g/day)	68.2 ± 34.7	71.7 ± 33.8	0.003	0.093	69.1 ± 35.1	73.0 ± 33.9	0.003	0.244	56.9 ± 27.2	48.7 ± 24.1	0.002	0.440
Fats (g/day)	31.1 ± 31.1	33.8 ± 24.3	0.008	0.334	31.7 ± 31.9	34.5 ± 24.7	0.007	0.345	23.5 ± 16.6	21.5 ± 10.1	0.068	0.754
CHO (g/day)	344.0 ± 114.0	326.6 ± 102.7	0.002	0.276	346.4 ± 115.5	329.1 ± 100.9	0.003	0.110	314.2 ± 88.8	280.4 ± 129.7	0.022	0.379
**Physical activity**				0.396				0.438				0.445
Insufficient, n (%)	353 (54.5)	23 (64.3)		0.022	202 (62.1)	10 (52.4)		0.015	176 (54.7)	10 (63.3)	0.131	
Sufficient, n (%)	295 (45.5)	12 (35.7)		0.022	124 (37.9)	9 (47.6)		0.015	146 (45.3)	6 (36.7)	0.131	
**Fall risk parameters**												
Fall experience, n (%)	161 (24.9)	9 (26.7)	0.009	0.878	73 (22.5)	4 (21.0)	0.128	0.491	81 (25.3)	3 (20.4)	0.025	0.703
Fall efficacy scale	94.6 ± 9.0	97.2 ± 10.2	−0.163	0.098	89.0 ± 14.1	88.2 ± 9.7	−0.628	0.744	90.0 ± 13.6	92.1 ± 9.7	0.064	0.440
ABC scale	80.5 ± 19.1	82.1 ± 16.0	−0.088	0.640	90.0 ± 7.9	95.8 ± 6.8	0.263	0.056	78.7 ± 19.3	76.5 ± 16.1	0.126	0.253
**Mental health parameters**												
K-MMSE score	25.2 ± 3.8	25.8 ± 2.8	−0.127	0.385	27.2 ± 2.1	26.7 ± 2.9	−0.137	0.449	24.8 ± 3.9	25.3 ± 2.7	−0.137	0.537
CESD−10 score	7.0 ± 8.7	8.1 ± 9.3	−0.156	0.532	6.5 ± 9.0	7.8 ± 11.7	−0.241	0.674	7.1 ± 8.7	8.3 ± 8.6	−0.129	0.572

ES: Cohen’s d; FBG, fasting blood glucose; TC, total cholesterol; TG: triglyceride; HDLC, high-density lipoprotein cholesterol; LDLC, low-density lipoprotein cholesterol; K-MMSE, Korean-mini-mental state examination; CESD-10, the 10-item short-form of the Center for Epidemiological Studies Depression; ABC, activities-specific balance confidence.

## Data Availability

The datasets used and analyzed in this study are available from the corresponding author upon reasonable request.
